# Journeying with developmental coordination disorder: The family experience

**DOI:** 10.4102/ajod.v12i0.1210

**Published:** 2023-12-19

**Authors:** Nicola L. O’Kelly, Jean V. Fourie

**Affiliations:** 1 Department of Educational Psychology, Faculty of Education, University of Johannesburg, Johannesburg, South Africa

**Keywords:** developmental coordination disorder, dyspraxia, neurodevelopmental disorder, family support, phenomenological study, qualitative research, family burden, occupational therapy, educational psychology

## Abstract

**Background:**

Developmental coordination disorder (DCD) is a neurodevelopmental disorder impacting 5% – 6% of children and continues into adulthood for 50% – 70% of cases. Despite the multidomain and lifelong influence of this disorder, little consideration has been given to the experiences of the family. Post-diagnostic support has been recommended however, the specific areas requiring support remain vague.

**Objectives:**

This study described the familial experiences of living with a member diagnosed with DCD.

**Method:**

A qualitative descriptive study using a phenomenological approach allowed insight into the lived experiences of families journeying with DCD. Forty-four participants representing 8 countries participated in an online questionnaire with 12 participating in an online semi-structured interview.

**Results:**

Themes generated reveal that obtaining a diagnosis and navigating the healthcare and education systems can be troublesome. Upon diagnosis, families tend to experience positive emotions such as relief. However, the daily challenges soon result in dominant negative emotional responses. DCD places significant financial burdens on families and impacts marital, parental and sibling relationships. Families often feel isolated from their communities as DCD is poorly understood.

**Conclusion:**

DCD places families at risk as daily struggles require support which targets identified motor, cognitive, academic and emotional challenges. Creating awareness in society, education and healthcare would alleviate continual frustrations.

**Contribution:**

This study provides insight into the wide-ranging impact that DCD has on families so that individualised support can be tailored, and general awareness raised.

## Introduction

Developmental coordination disorder (DCD), often referred to as dyspraxia, is a neurodevelopmental disorder (American Psychiatric Association [APA] 2022). Despite the 5% – 6% prevalence among school-going children (APA 2013), children with DCD often find themselves overlooked or misdiagnosed (Farmer, Echenne & Bentourkia 2016; Missiuna et al. 2007). Although DCD is classified as a neurodevelopmental disorder, it is estimated that 50% – 70% of children with DCD continue to experience difficulties into adolescence and adulthood (Blank et al. 2019). The aetiology of this disorder remains unclear (Brown-Lum & Zwicker 2015).

Developmental coordination disorder was first included in the Diagnostic and Statistical Manual of Mental Disorders (DSM) in 1987 (DSM-III-R) (APA 1987). The diagnostic criteria were refined in 1994 (DSM-IV) and again in the DSM-5 in 2013. The current diagnostic criteria include challenges with the acquisition and execution of motor skills that are well below what would be expected, given the individual’s chronological age and the provision of opportunity to develop the skills. These difficulties with motor coordination significantly and persistently interfere with activities of daily living, academic or vocational productivity, leisure and play. The DCD is not acquired but rather, the challenges can be tracked back to the early developmental period. It is a diagnosis of exclusion in that the motor skills deficits are not better explained by an intellectual disability, visual impairment or other neurological conditions (APA 2022).

The DCD has a multidomain impact (Meachon 2023) where fine and gross motor, cognitive and academic, social, emotional and behavioural challenges may be present (Winson 2018). Fine motor challenges may result in difficulties with dexterity, hand-eye coordination and balance (Prunty et al. 2016; Sadock, Sadock & Ruiz 2015). Challenges with activities such as handwriting, cutting, eating with utensils, fastening buttons and tying shoelaces may be evident (Case-Smith 2005; Farmer et al. 2016). Deficits with motor coordination may impact speech (Gaines & Missiuna 2007) and result in ophthalmological abnormalities, for example, difficulty in tracking text across a page (Creavin et al. 2014; Rafique & Northway 2015). Challenges with gross motor coordination may result in difficulties perceiving, sequencing, planning and actioning movements, which may cause clumsiness (Kranowitz 2005). Maintaining an appropriate posture and participating in sporting activities is difficult (Speedtsberg et al. 2017). The motor skill deficits may negatively impact the individual’s academic performance and social, emotional and behavioural functioning.

Fine motor challenges, especially those associated with handwriting and speech, may negatively impact the individual’s academic output and may result in the presentation of written or spoken work, which is not an accurate reflection of the individual’s cognitive capabilities (Prunty et al. 2016). Children with DCD tend to experience greater challenges with executive functioning when compared with typically developing peers (Bernard et al. 2018; Lachambre et al. 2021; Meachon, Zemp & Alpers 2022). Concentration challenges may also be present and appear to be related to the amount of energy required for gross and fine motor control (Cermak & Larkin 2002; Dewey et al. 2002; Farmer et al. 2016).

Individuals with DCD seem to be at greater risk of psychosocial challenges. This is because of DCD acting as a primary stressor, which places the individual at risk of exposure to secondary stressors as a result of interpersonal and intrapersonal difficulties (Blank et al. 2019; Meachon et al. 2022; Sadock et al. 2015). Children with DCD may find tasks challenging, which their peers find easy. As a result, each school day may be filled with frustration and disappointments, which culminate in emotional outbursts (Missiuna et al. 2007). Teachers identified children with DCD as displaying more emotional and behavioural challenges when compared with their typically developing peers (Crane, Sumner & Hill 2017; Van den Heuvel et al. 2016). Heightened levels of anxiety may also exacerbate the motor challenges (Harris, Purcell & Wilmut 2022). As a result of the deficits applying motor and verbal skills, the playing of games is challenging, which negatively impacts the individual’s ability to participate in social leisure activities (Winson 2018). Children with DCD tend to rely less on technology to socialise with peers (Izadi-Najafabadi et al. 2019).

Although DCD itself does not cause social difficulties, the interpersonal challenges experienced over a prolonged time may result in decreased self-competence, anxiety and depression (Saban & Kirby 2019). The impact of DCD on the individual has been studied and little consideration has been given to the influence of DCD on the family (Stephenson & Chesson 2008) despite the challenges impacting the individual also affecting the family unit (Blank et al. 2019; Cleaton, Lorgelly & Kirby 2019; Stephenson & Chesson 2008). Even before embarking on the diagnostic journey, parents may begin to recognise subtle differences between their child and typically developing peers (Missiuna et al. 2006). Unfortunately, when these suspicions are shared with medical professionals, they are often dismissed (Missiuna et al. 2006). General awareness of clinicians and experience in working with DCD is low when compared with ADHD (Meachon, Melching & Alpers 2023).

This may be exacerbated by the various scopes of practice, which allow for only some healthcare professionals, medical doctors and psychologists to diagnose DCD, while others such as speech and occupational therapists are not allowed to diagnose (Department of Health [DoH] 1974, 2017, 2021; Health Professions Council of South Africa [HPCSA] 2019). However, even when a DCD diagnosis has been obtained, attempts to activate support, especially within the education systems, may be challenging (Missiuna et al. 2006; Winson & Fourie 2020). Advocating for a family member with DCD can be a time-consuming process (Missiuna et al. 2006). As a result, primarily mothers may be faced with the decision to reduce their work hours or transition to a different career path with more flexibility to support their child with DCD (Cleaton et al. 2019). Assisting family members with motor tasks can also be time-consuming (Stephenson & Chesson 2008). Parents may find it difficult to identify when to provide the child with DCD the opportunity to attempt the motor skill themselves or when they should support the child in the process (Missiuna et al. 2006). A study that considered mothers of children with DCD found elevated levels of worry, stress, anger and frustration resulting in emotional fatigue (Cleaton et al. 2019). However, fathers and siblings are also influenced (Stephenson & Chesson 2008). Siblings may receive less parental time and attention and may experience frustration, jealousy, embarrassment or worry (Cleaton et al. 2019). The individual with DCD and the family unit participate less in social activities. Up to a third of families journeying with DCD find the DCD impacts on their family activity choices, holiday plans and social gatherings (Cleaton et al. 2019).

A parent organisation participating in the development of the Clinical Practice Recommendations on the definition, diagnosis, assessment, intervention and psychosocial aspects of DCD identified five key areas, which would be beneficial to families. These areas included greater awareness and recognition of DCD by healthcare professions, the education system and public; improved access to services; a clear diagnostic pathway; greater information regarding therapeutic options and the effectiveness of these options (Blank et al. 2019).

The multidomain and lifelong impact of DCD influences not only the individual diagnosed with the condition but also the family unit. Therefore, this study explored the familial experiences of journeying with DCD.

## Research method and design

A qualitative descriptive study using a phenomenological approach was utilised in order to gain greater understanding of the lived experiences of families journeying with DCD. Purposeful sampling took place. Parents raising children with DCD were included in the study as were adults with DCD who were able to reflect on their family’s journey with DCD. Participants were not excluded if their family structure did not conform to historical views of a typical family nor were participants excluded based on the presence of co-occurring conditions.

The data collection phase of this study took place during the coronavirus disease 2019 (COVID-19) global pandemic while restrictions were placed on personal interactions. As a result, participants were recruited through online means. After ethical clearance was gained from the University of Johannesburg, Research Ethics Committee, an online advertisement requesting participants was placed on six social media groups, which specified that they were related to DCD or dyspraxia. The participant recruitment advertisement was shared with two social media groups associated with allied health professionals and psychologists in South Africa. The online research participant advertisement included the background to the research as well as a QR code and live link to the online questionnaire. A total of 44 participants from 8 countries responded to the recruitment and completed the online questionnaire. Countries represented were South Africa (*n* = 6), the United Kingdom (*n* = 14), the United States (*n* = 12), Canada (*n* = 4), Australia (*n* = 3), New Zealand (*n* = 1), Egypt (*n* = 1) and Indonesia (*n* = 1).

The questionnaire contained both forced-choice and open-ended items. The forced-choice items included questions pertaining to demographic information, the nature of the DCD within the family, the type of healthcare professional through whom a diagnosis was obtained, and the level of expertise observed in the healthcare professional. Open-ended items included a description of the diagnostic process, the impact of the DCD on different family members, and the skills required to navigate the journey. Participants who after completing the questionnaire desired to share further insight into their family’s journey with DCD were asked to leave their contact details so that an in-depth interview could be arranged, and 12 participants agreed to participate. The initial analysis of the online questionnaire questions provided the opportunity to make use of the semi-structured online interview to probe further issues raised through the analysis of the online questionnaires. This allowed for some key questions to be asked during all interviews as well as to ask individualised questions. The online interviews lasted approximately half an hour each. The 12 participants were numbered P1–P12 based on the order in which the interviews were conducted. An ‘i’ was used to distinguish the interview information from the questionnaire data ‘q’ during the data analysis process. The additional 32 participants were numbered P13–P44. All the 12 participants who participated in the questionnaire and interview were diagnosed with DCD, 8 had a biological son with DCD, 1 had a biological daughter with DCD and 2 had DCD themselves. One family had two members diagnosed with DCD. Of the additional 32 participants who participated in the questionnaire, 26 had received a DCD diagnosis, 9 suspected DCD, 12 had biological sons with DCD, 6 biological daughters and 1 adopted daughter had DCD, and 2 fathers. Twelve individuals had DCD themself. Three of the families had more than one member with suspected or diagnosed DCD.

During the data analysis phase, themes were developed and meaning sought from the raw data (Mann 2016). The data analysis process progressed through five stages (Yin 2016), which took place in a cyclic rather than a linear manner (Taylor et al. 2016). The data analysis process began as soon as the data were collected. Questionnaires completed via Google Forms were stored in Excel format while the online interviews were audio-recorded and transcribed verbatim. The open-ended items from the questionnaires and the transcribed interviews were uploaded into ATLAS.ti (https://atlasti.com/) to assist with the disassembly and coding. Codes such as ‘diagnostic process’, ‘emotional impact’ and ‘siblings’ were used to identify patterns in the data so that the unique viewpoints of the participants could be contrasted and compared (Richards 2015). The data, disassembled into coded units, were then reassembled into themes (Yin 2016). The coded data from each participant were pooled together according to code to analyse and synthesise the subjective views and experiences shared by the participants (Morse 2017). This allowed for the interpretation of the data to occur.

### Ethical considerations

This study was approved by the University of Johannesburg, Faculty of Education Research Ethics Committee (NHREC No.: REC-110613-036) with ethical clearance number 2020019. A participant information sheet that included the background to the study, aims of the research, participant selection requirements and information about the researcher was produced and shared with participants. The participants were made aware of their voluntary participation in the study as well as their right to withdraw from the study at any time. Audio-recorded verbal consent was obtained from participants in the online interviews, and written consent was obtained for those participating in the questionnaire. A pseudonym was used to protect the identity of each participant.

## Results

**FIGURE 1:**
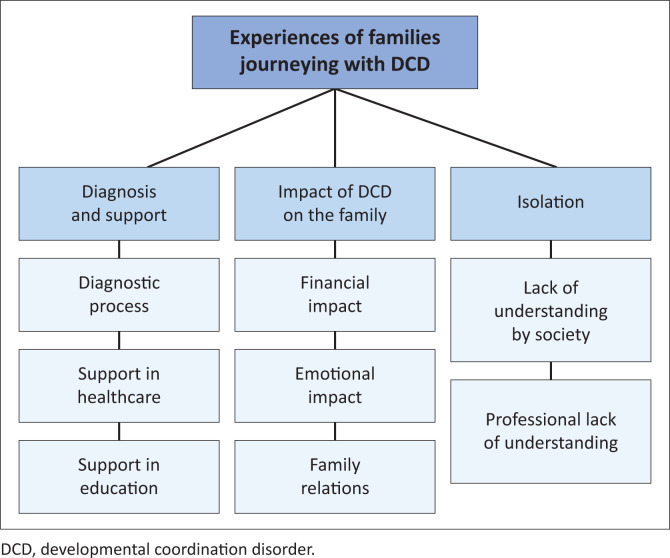
Experiences of families journeying with developmental coordination disorder.

Three main themes concerning the familial experience of journeying with DCD emerged through the data analysis (Figure 1), namely, difficulties experienced during the diagnosis and search for support, the specific impact of the journey with DCD on the family unit, and a sense of isolation.

### Theme 1: Diagnosis and support frustrations

Family members usually identified symptomatic difficulties their child was experiencing before embarking on the diagnostic journey. Families saw delayed milestones, challenges with fine and gross motor skill development, delayed speech development or clumsiness as the first signs of concern. However, once these concerns were raised, a common theme was ‘the experience of frustrations in the diagnostic process’. The length of time taken to obtain a diagnosis ranged from 2 to over 7 years – 2 years for 45% of the participants; 21% of the participants were only diagnosed after 5 years or longer. The length of time taken to obtain a diagnosis appeared to be related to the healthcare professional’s knowledge of DCD. One family explained that:

‘It’s bizarre to me, because we had been seeing medical professionals, since [child with DCD] was five, people who should have known. I mean, I diagnosed him off information from the internet, when he was probably seven. And then it still took another five years because they wouldn’t listen.’ (P6i1, female, USA)

Another parent simply stated that, ‘The health professionals have got no clue’ (P8i31, female, Canada). Many families found their concerns being dismissed by healthcare professionals. ‘I asked our paediatrician about it …and he kinda blew me off. He was like, “You know he’s just a laid-back kid”…’ (P1i5, female, USA). ‘So initially, they wrote it off to the fact that he is a boy, he’s a bit slower, etc.’ (P3i2, female, ZA). This often resulted in families feeling unheard and unsure of the next steps to take.

A parent complained that ‘No one was making referrals. No one thought there was an actual diagnosable problem … only that he was “delayed” and would eventually catch up’ (P6q3, female, USA).

Another parent added that:

‘There seem to be few or no local experts with specific insights into the condition, so it was always a case of taking one suggested diagnosis at a time and seeing if that fit symptoms and behaviour, rather than having the condition clearly diagnosed and given steps to move forward.’ (P41q1, male, ZA)

In addition to the frustration, parents often experienced the diagnostic process to be a period of immense stress and worry. One family stated that, ‘One of the worst feelings is knowing there is something not right with your child and, despite your best efforts, remaining undiagnosed, I worried a lot’ (P21q3, female, Australia).

Parents were often unsure of which healthcare professional to consult. For example, ‘I wish we were sent to the developmental paediatrician first. We went to a genetics doctor first, then on our own started OT who recommended seeing the developmental paediatrician’ (P43q9, female, USA). Another parent described the diagnostic process like ‘going from A to Z to get to B’ (P4i21, female, UK).

Parents pursued the diagnostic process in the hopes that obtaining an official diagnosis would lead to the provision of much required support. However, although some families received support, the majority were unsupported as a parent stated, ‘I received a report and that was it’ (P17q2, female, UK). After receiving a diagnosis of DCD, families often found themselves having to identify the relevant support for themselves and approach the various healthcare professionals. ‘We have independently sought help from therapists, physio [physiotherapist], OT [Occupational Therapist], Speech, Audiology, Psychology’ (P29q10, female, ZA).

Navigating the diagnostic process takes a toll on the family with one parent explaining:

‘A lot of these medical professionals focus on the patient but fail to take into account the family environment and what impact this has on the patient, as well as the impact of the patient’s disability on the family’ (P2q14, female, AU).

In addition, parents also often find themselves having to educate the healthcare professionals on the condition of DCD. One parent explained that:

‘After I got the DCD diagnosis from the behavioural therapist, I went to the paediatric neurologist. I needed to get a letter for the school to officially give him the diagnosis. I went in and I talked to the guy, and I told him, this is what I need to give to the school so we can have accommodations and it can be documented. And he says, “What’s DCD?” I say, “Developmental Coordination Disorder”. He said, “That’s not a thing. That’s not a thing”. I was like, “It’s in the DSM 5”. I had to show him the pages. I had the pages with me’ (P6i26, female, USA).

Another parent explained that, ‘The amount of medical professionals I have told, “[child with DCD] has got DCD”, “Oh, what’s that?” And I think well, shouldn’t you know that?’ (P2i7, female, AU). Instead of practitioners offering support and understanding to families, parents are educating the practitioner.

Furthermore, families also encounter hurdles while attempting to obtain support in the education systems. ‘Even with the diagnosis the schools will very often tell you, “We won’t let them have special education or any accommodations”’ (P6i44, female, USA). These challenges with obtaining support from school tend to be associated with a lack of understanding. ‘It is not a well understood disorder, especially in schools’ (P3q11, female, ZA).

Although the diagnostic criteria for DCD (APA 2022) allude to academic challenges, focus tends to be placed on the motor coordination difficulties resulting in families finding difficulty obtaining the necessary support regarding schoolwork:

‘It was hard to make it clear with teachers and … with the school especially, that the help he needed was not just in PE with his motor skills.’ (P5i11, female, USA)

Another parent opined:

‘It was a struggle to get the IEP because they didn’t really associate it with things they can help with at school. It was like, “you have outside occupational therapy, so what do you want us to do?”’ (P5i9, female, USA).

The challenges faced by individuals with DCD in the classroom may reduce the opportunities for reaching their academic potential, ‘Academically my son is gifted. But he cannot go to a school (for the gifted) because of his challenges. This frustrates him immensely’ (P32q4, female, ZA).

Some parents reported that the school did not believe the DCD diagnosis, ‘The school didn’t believe me or the OT that my daughter has dyspraxia’ (P16q, female, ZA). A parent explained DCD to the teachers, advocating on behalf of the child and even providing the support suggestions:

‘None of them (teachers) had any idea what dyspraxia was. A lot of times they were like, “Oh, you mean dysgraphia?” Well, no… Once I explained that it was more motor, and not just the idea of nice handwriting. Once they saw him in their classroom, and realised that it wasn’t him being lazy, he just couldn’t do it they were a little more understanding, but I was still the one who had to do the research behind it, and then give them all the prompts like, “Hey, this is what we do at home?” Or “How about you try this? This is what worked last year”’ (P7i7, female, USA).

Another parent explained:

‘The majority of teachers have no clue what it is, absolutely no idea. And I have to provide them with information about DCD. Even resource teachers when it first was diagnosed, I had to get information online. I gave them websites that they could access to know what it was and what it meant for them in the classroom.’ (P11i1, female, CA)

Having identified their child’s symptoms associated with DCD, many families found the diagnostic process time-consuming and frustrating. Thereafter, acquiring of relevant support from healthcare and education practitioners required continual advocacy and explanation.

### Theme 2: Financial, emotional and relational stress

Participants identified a wide-ranging impact of DCD on the family unit with significant financial stress, time spent, emotional strain and impacts on family relations.

While some of the participants live in countries where education and healthcare are free, for 21 of the 44 participants, DCD and the support required as a result of the associated challenges has been a significant financial burden for the family. The cost of the supportive interventions and the extended amount of time required for implementation was reported, ‘Financial (cost) has been the biggest impact’ (P5q6, female, USA). ‘We’ve spent thousands on OT and Speech for over a decade’ (P12q9, female, ZA). A mother from Australia added that, ‘With paediatric physio, OT, behavioural optometrists, paediatrician, speech pathologists – it cost a lot of money for about 10 years’ (P20q11, female, AU).

Some families were in the fortunate position to have a parent primarily staying at home and able to provide the focused time required; for example, ‘I’m mostly a stay-at-home parent so most of the appointments and working with the IEP (Individual Education Plan) team and all of that has been me’ (P5i28, female, USA). Others have chosen their career paths in order to have the flexibility to be able to provide the necessary support, ‘I’ve always structured the kind of work I’ve done around my kids’ (P8i13, female, CA). However, this can further impact the financial burden experienced by families as the amount of work hours available is decreased. Some families indicate that the ability of a parent to work has been reduced by the time spent at consultations. ‘Therapy sessions are multiple times per week impacting my ability to work’ (P21q10, female, AU), while another stated that, ‘It has impacted me professionally, not able to work as much while trying to find help and has impacted our family financially’ (P39q4, female, USA). Other families have chosen to self-support a child, which is also time-consuming and affects the availability to work, ‘Financially we are committed to home-schooling… so that is a real struggle with one income’ (P28q5, female, NZ).

With the provision of financial support through healthcare and education, some countries have mitigated the financial burden experienced by families journeying with DCD; however, the emotional impact of this disorder on the family is draining. One mother explained that:

‘As a mum with a family that is not coping with [child with DCD’s] needs, I feel like we’re doing this solo. While the funding is amazing, I just want someone to hold my hand and help me along this extremely difficult journey.’ (P2i10, female, AU)

**FIGURE 2:**
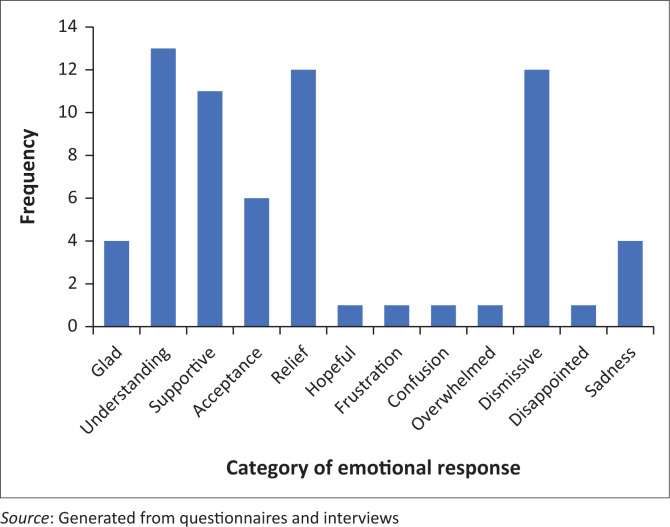
Frequency of Emotional responses to the diagnosis of DCD.

A variety of emotional responses to the DCD diagnosis were shared by the participants (Figure 2). Frequently, the diagnosis was met with a positive response such as understanding, relief and acceptance. The greatest negative emotion shared was associated with family members dismissing the severity of the diagnosis.

The positive emotional responses to the diagnosis may result from the frustration and stress experienced during the diagnosis process, for example, ‘We were glad to have a diagnosis that might help inform our path to supporting our son’ (P1q2, male, USA). The positive response to the diagnosis tended to be associated with immediate and maternal family members, ‘My mother was relieved because my struggles could finally be attributed to a specific condition’ (P9q2, female, USA). While fathers and extended family tended to have higher levels of negative emotional response to the diagnosis of the condition, ‘Father doesn’t really care. That’s a man thing’ (P18q4, female, CA), ‘Mother: accepting. Father: denial and doesn’t understand the illness’ (P31q2, female, Egypt), ‘My husband doesn’t really want to believe it as my daughter does extremely well≈academically’ (P16q3, female, ZA), and ‘Grandma dismissive, believes it’s a label for the sake of a label’ (P10q3, female, UK).

Although generally positive rather than negative emotional response to the diagnosis was identified, some families may experience concern regarding the negative impact that a diagnostic label may have on the family member. For example, ‘All (parents and sister) with mixed relief having something to work with going forward and trepidation as to the limits the condition places on those who have it’ (P41q20, female, ZA). The generally positive response to the diagnosis may be attributed to the active pursuit of the diagnosis by the participants in this study with the hope that a diagnosis may result in support.

**FIGURE 3:**
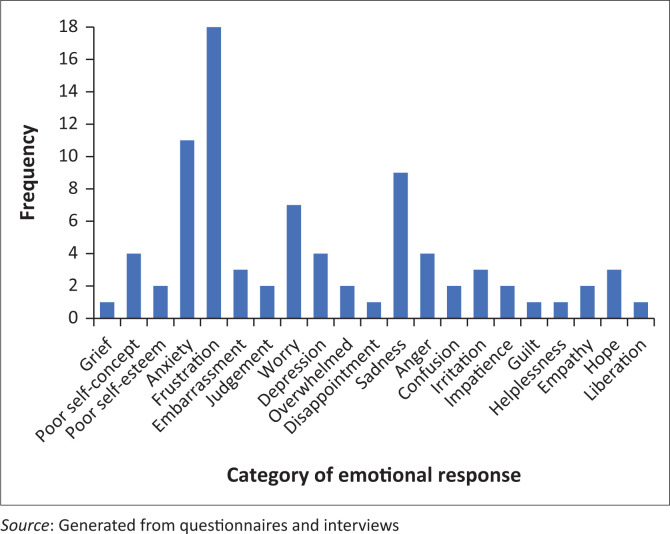
Frequency of Emotional responses on the impact of DCD on the family.

However, as the families journeyed on from the diagnosis, understanding, relief and supportiveness were replaced with frustration, anxiety and sadness during the day-to-day living experiences (Figure 3). The development of empathy, hope and a sense of liberation were overshadowed by worry, depression and poor self-concept. This may be associated with the realisation that the diagnosis did not provide the level of support hoped for or the realisation of the multidomain and lifelong impact of this condition on the family.

As the family navigates daily life with DCD, the realities of the lifelong difficulties become apparent resulting in grieving emotional responses. One mother explained:

‘There’s this real grief of like, I don’t have this, normal neurotypical kid, I have this little bundle of something called … complexity. And that just means that I have to adapt, and that’s hard.’ (P2i26, female, AU)

As the journey with DCD continues, families see the developmental delays between their child and the peer group widening, as a parent observed:

‘Friends two years his junior and younger are overtaking him in some spheres and now commenting on his functioning, and it hurts to see him beginning to stand out more because of his delays.’ (P12q8, female, ZA)

Another parent lamented that, ‘It’s hard to watch his younger brother easily do things he struggles with’ (P43q5, female, USA). For individuals with a DCD diagnosis, their self-esteem and self-concept were influenced, as a participant stated, ‘I grew up believing I was broken and weird. Teachers, family, and my peers never expected me to achieve anything’ (P4q2, female, UK).

Another participant explained that DCD was the root cause of their challenges with mental health:

‘I will say it’s had a significant impact on my mental health because I never had a lot of self-esteem. And I think a lot of that I can attribute to the DCD because I struggled in school, and I never felt like I belonged or was intelligent enough to go to university, or to even be in main line high school. And then I also struggled with employment, and it takes a toll on your self-esteem…. And while I don’t want to say it’s all because of DCD, I feel like that kind of underpins all of my self-esteem issues.’ (P9i41, female, USA)

Parents also observed their children’s mental health difficulties, ‘He thinks he’s dumb, stupid, weird and no one wants to be his friend. This isn’t true but it’s his perception’ (P6q16, female, USA). Another mother added:

‘Through the years with the hardship of school, being picked on in PE lessons, always coming last at everything he does, it takes a toll. He is 18 and is now clinically depressed and on medication.’ (P20q9, female, AU)

A parent expressed, ‘I get angry with myself for losing my patience and temper with him when his difficulties make situations stressful’ (P12q7, female, ZA).

One mother highlighted that DCD is only one of the challenges faced by the family:

‘I think I’m still in the survival stages. And that might be more than just the DCD, you know, when you add all the different balls that I’m kind of juggling for the whole family. That’s where I think my daily survival is kind of coming from.’ (P2i27, female, AU)

Another mother commented that she experiences ‘emotional challenges particularly around trying to balance the siblings, and my anxiety’ (P29q8, female, ZA).

Behavioural challenges associated with schoolwork were reported, ‘He has difficulties with reading and writing especially… So, when school started again, he started acting out’ (P5i7, female, USA).

The DCD had some positive influences as one participant explained:

‘I know that I wouldn’t be who I am and I wouldn’t have the insights I have without my dyspraxia, and I wouldn’t be able to think about things and come up with solutions if I was neurotypical. So, I’ve actually found myself quite grateful that I am. Despite all the challenges, I am finding that I don’t actually think I would swap it.’ (P4i25-26, female, UK)

One participant, although not diminishing the challenges, highlighted the positive aspects of living with DCD, ‘Living with this sucks, but I feel like it has made me a very empathetic person and it has contributed to my creativity’ (P23q8, female, USA).

Parenting a child with a disorder that is generally not well understood by healthcare or society can result in self-doubt regarding the ability to parent effectively. One family explained, ‘You start to feel like a bad parent. “What am I doing wrong? Why isn’t this working?” (P6i8, female, USA).

One parent shared that the family was now a closer unit:

‘That was really stressful because my husband didn’t understand at that point. But I think now, we’re probably closer for it because we’re a team and we’re working with my son, to help him grow and to help him learn, and we work on strategies together.’ (P11i30, female, CA)

Another mother explained:

‘I think my biggest challenge with his father is he was a sportsman … He wants his son to do rugby, he wants his son to do this, that, and the next thing … He wants this perfect son that can follow in his footsteps, and he can’t actually have it … That does also put a strain on the relationship because you have to acknowledge that he’s got special needs that we have to take care of.’ (P3i34-40, ZA)

The importance of speaking openly about DCD as a family was mentioned, ‘I think it would be very bad for the whole family dynamic if we weren’t talking about it’ (P6i32, female, USA).

The DCD influences the everyday experiences in which the family engages as parents explained, ‘We rearrange our family trips and outings based on abilities at the time’ (P7q6, female, USA); ‘Socially it limits how much we can do without overwhelming our son’ (P28q4, female, New Zealand); and ‘Outings are limited by the physical disability. We cannot hike as a family and when traveling, assisted passage must always be booked for airports’ (P29q6, female, ZA).

The sibling relationship and relationship between typically developing siblings and parents can also be impacted by DCD. A parent family explained that ‘therapy and doctor visits are very time-consuming, and the older sister feels neglected’ (P29q5, female, ZA). Some families recognise the amount of attention given to family member with DCD but attempt to provide time for each family member and the family as a unit:

‘Invariably he [sibling with DCD] does get a lot of extra attention. So, we try to balance it out. She’s [neurotypical sibling] got things that she does. He’s got things that he does and then there are things we try and do together.’ (P5i25, female, USA)

While some siblings readily accept their sibling with DCD and may even appear to not notice the condition, others experience a range of negative emotions. For example, ‘Frustration and anger from his sister as she perceives him to get special/more lenient treatment from us as parents’ (P12q3, female, ZA). Another family commented on the frustration and irritation experienced by an elder sibling:

‘Even though she understands his limitations, she still gets frustrated and mad that he “gets away” with things that I would never have let her “get away” with. She also gets irritated that I don’t make him do the same level or number of chores that she has.’ (P6q9, female, USA)

A parent explained the sibling’s emotional experiences:

‘My younger daughter gets upset when [child with DCD] gets sad when he is struggling with something. He is 18 and struggling to learn to drive. My daughter is 16 and will start soon but she doesn’t want to start until he has his driver’s license. She doesn’t want to get it before he does.’ (P20i5, female, AU)

Worry can also be experienced by siblings, as a parent noted, ‘My daughter has had to grow up fast and is always worried about him’ (P20q7, female, AU).

The DCD can impact all areas of family functioning from ‘Getting ready for anything on time’ (P2q7, female, AU) to difficulties with organisation as the individual tends to ‘create chaos and mess everywhere’ (P36q4, female, UK). A participant summed up the impact of DCD on family life simply as, ‘We are being challenged in every aspect of life’ (P24q3, female, USA).

The DCD influences the family’s financial, emotional and relational well-being with both positive and negative aspects reported.

### Theme 3: Isolation

Many participants indicated a sense of isolation related to the lack of understanding of this condition within healthcare, education and society in general. Families may find themselves isolated from social settings as a parent explained, ‘Socially we are somewhat isolated because people don’t understand [child with DCD’s] unique personality and the physical challenges he lives with every day’ (P6q11, female, USA).

Another parent explained that the DCD symptoms can be misinterpreted by family members and friends, ‘Friends and family often ascribe his difficulties to permissive and bad parenting’ (P12q11, female, ZA). The social isolation because of a lack of social understanding may in turn exacerbate the emotional challenges experienced by the family.

As a result of the lack of understanding of DCD in society, families often have to explain the condition to others which is challenging, ‘I think that’s probably one of the biggest challenges we have is trying to actually communicate what he has to others’ (P3i44, female, ZA). Another parent added that, ‘It’s hard to explain to people who don’t understand’ (P9i16, USA). The ‘invisibility’ of the disorder contributes to difficulties providing explanations, ‘My family had never heard of DCD so trying to explain an invisible thing is quite hard to do’ (P20q4, AU).

Increased awareness would support families in being confident to participate in society without fear of rejection or misunderstanding:

‘I would like to have more social awareness of this. Often in our social circles people expect my son to behave as typical children do, and when he gets frustrated, they often perceive this as misbehaviour.’ (P32q8, female, ZA)

For some families, the lack of understanding can result in envy for those conditions, which carry greater awareness, ‘I used to almost wish he was autistic because there’s like tons of support for that and tons of reading and tons of information’ (P1i48, USA). Another parent explained that ‘Autism, they understand, they’ve seen it in the movies, dyspraxia they haven’t yet. So that’s a challenge’ (P3i44, female, ZA).

A participant pointed out that the lack of understanding of DCD by healthcare professionals delegitimises the disorder:

‘I feel like it kind of like delegitimises it, when you have the diagnosis of autism, or even ADHD, they know about it, and they have services established for it.’ (P5i13, female, USA)

## Discussion

Developmental coordination disorder is a pervasive condition that influences every aspect of the individual’s life and the family unit, thus greater awareness of DCD, and an understanding of the family’s everyday experiences could assist healthcare and educational professionals to better support families journeying with this condition. This study highlighted the difficulties of obtaining a clear diagnosis and then navigating the healthcare system. These difficulties were commonly reported in first world and developing countries. In this study a parent from Australia reported that although medical funding was available, she would have appreciated support along the difficult way. South Africa has public and private medical facilities, however parents still struggled with the diagnosis visiting various medical professionals, such as the general doctor, physiotherapist, occupational therapist, speech therapist, audiologist and psychologist. Although all health professionals should know the diagnostic criteria of DCD, the condition’s motor coordination difficulties are often subtle in their manifestation and thus not overtly obvious in the consultation room. Even though the symptoms are visible if practitioners know what to observe, the symptoms are often dismissed or overlooked. South African parents in this study could afford access to the private healthcare system, but there was no pathway with clearly defined symptom recognition for the practitioners to follow. We surmise that difficulties with the diagnostic process will be exacerbated for the majority of parents in South Africa who only have access to the public primary healthcare system. The length of time spent in search of a diagnosis was similarly reported by Soriano, Hill and Crane (2015) who found that, on average, a DCD diagnosis was confirmed after two and a half years. A lack of understanding during the diagnostic process was similarly reported by the participants in the study by Missiuna et al. (2006). In addition, 43% of the families in Soriano et al.’s (2015) study were not offered any practical support during the diagnostic process. As in this study, these healthcare professionals tended to minimise the concerns described by the parents with the assumption that the child would outgrow the challenges.

In this study, challenges navigating the education system were also raised. Parents reported that few teachers in schools are aware of the condition, and even with a clear diagnosis, one parent in South Africa reported that the school would not believe the diagnosis. Parents then inform teachers of their child’s condition and mobilise supportive interventions as found in a similar study by Missiuna et al. (2006). Developmental coordination disorder affects planning of schoolwork (Kranowitz 2005), and the symptoms related to impaired fine motor coordination influence skills of manual dexterity related to writing, cutting, dressing, doing puzzles and playing sport which are onerous and frustrating to execute quickly and neatly (Winson & Fourie 2020).

Upon diagnosis, families tend to experience positive emotions such as relief, understanding and acceptance. However, as the journey with the disorder continues the daily challenges realised, a negative emotional response tends to develop with frustration, sadness and anxiety overshadowing the previous positive emotions. With the high levels of negative emotion experienced by families, it is unsurprising that Cleaton et al. (2019) found that nearly three-quarters of the participants were at risk of depression.

In addition to the emotional response, families are often faced with the financial burden of paying out of pocket for supportive interventions and specialised schooling. The time spent in doctors’ appointments, therapies and support can reduce the availability of a parent to participate in the workforce creating a further financial burden. These time constraints result in reduced capacity for a parent to follow a career of their choice as previously reported (Cleaton et al. 2019; Stephenson & Chesson 2008). A parent in South Africa reported spending thousands on medical interventions in the past decade. It is unfortunate that only a minority of South African parents could afford such expenditure, whereas most parents would be reliant on accessing the overburdened public healthcare system.

The challenges faced as a family can influence the relations between parents and siblings. Similarly, Stephenson and Chesson (2008) found that while some families experienced marital breakdown, others grew closer together. Challenges faced by siblings have been previously identified (Cleaton et al. 2019). Although the disorder can unify a family, it can also contribute towards conflict, jealousy and misunderstanding. Finally, families often feel isolated from their communities and social sphere because of a general lack of understanding of DCD. Families are often faced with explaining the condition to the public. This sense of isolation was attributed to a lack of social awareness and the avoidance of certain activities. Similarly, Cleaton et al. (2019) reported that a third of the participants made changes to family activities to accommodate the family member with DCD.

Further research into the specific support that can be provided to parents is required to better support these families, particularly regarding the unequal public and private healthcare and education systems in South Africa.

## Recommendations and conclusion

This study highlights the significant impact that DCD has on the family unit despite only one member of the family being diagnosed with the condition. Healthcare professionals and educators should consider the individual with DCD within the context of their family so that holistic support can be provided. This may include the provision of location-specific, practical support suggestions for the family, options of family-based and individual therapy for the different members of the family to process their emotions and responses to the diagnosis of DCD. The training programmes of healthcare and education practitioners should include DCD in the curriculum. The development of protocols with clear pathways for diagnosis and post-diagnostic support could be developed to assist families with the diagnostic frustrations.

Healthcare professionals should make relevant referrals and work in multidisciplinary contexts, which include occupational therapists and educators so that families can be effectively guided through both the healthcare and education systems. Education and developing awareness of this ‘invisible thing’ would greatly facilitate integration and acceptance for families journeying with the frustrating condition of DCD.
